# RNA-Seq of Early-Infected Poplar Leaves by the Rust Pathogen *Melampsora larici-populina* Uncovers *PtSultr3;5*, a Fungal-Induced Host Sulfate Transporter

**DOI:** 10.1371/journal.pone.0044408

**Published:** 2012-08-30

**Authors:** Benjamin Petre, Emmanuelle Morin, Emilie Tisserant, Stéphane Hacquard, Corinne Da Silva, Julie Poulain, Christine Delaruelle, Francis Martin, Nicolas Rouhier, Annegret Kohler, Sébastien Duplessis

**Affiliations:** 1 Unité Mixte de Recherche 1136 ‘Interactions Arbres/Microorganismes’, INRA (Institut National de la Recherche Agronomique)/Université de Lorraine, Centre INRA de Nancy, Champenoux, France; 2 CEA-Genoscope, Centre National de Séquençage, Evry, France; Nanjing Agricultural University, China

## Abstract

Biotroph pathogens establish intimate interactions with their hosts that are conditioned by the successful secretion of effectors in infected tissues and subsequent manipulation of host physiology. The identification of early-expressed pathogen effectors and early-modulated host functions is currently a major goal to understand the molecular basis of biotrophy. Here, we report the 454-pyrosequencing transcriptome analysis of early stages of poplar leaf colonization by the rust fungus *Melampsora larici-populina*. Among the 841,301 reads considered for analysis, 616,879 and 649 were successfully mapped to *Populus trichocarpa* and *M. larici-populina* genome sequences, respectively**.** From a methodological aspect, these results indicate that this single approach is not appropriate to saturate poplar transcriptome and to follow transcript accumulation of the pathogen. We identified 19 pathogen transcripts encoding early-expressed small-secreted proteins representing candidate effectors of interest for forthcoming studies. Poplar RNA-Seq data were validated by oligoarrays and quantitatively analysed, which revealed a highly stable transcriptome with a single transcript encoding a sulfate transporter (herein named PtSultr3;5, POPTR_0006s16150) showing a dramatic increase upon colonization by either virulent or avirulent *M. larici-populina* strains. Perspectives connecting host sulfate transport and biotrophic lifestyle are discussed.

## Introduction

Parasitic microbes continuously challenge plants but constitutive and inducible host immunity is generally sufficient to confer broad-spectrum resistance to non-adapted pathogens [1]. A successful infection requires that microbes secrete effectors into infected tissues to repress and manipulate host defense and physiology, resulting in the promotion of biotrophic growth and the proper setting of compatible interaction [2]. In turn, plants possess several hundreds of resistance (R) proteins that, upon direct or indirect recognition of pathogen effectors, trigger strong defense responses and lead to incompatibility [3]. The issue of an interaction as compatible or incompatible is determined within hours following the formation of the first structures of infection and subsequent effector secretion, even after the very first physical contacts [4]. At these early stages, the ability of pathogen effectors to rapidly and efficiently manipulate host functions and simultaneously escape R protein recognition is though to be the key of compatibility [5]. Identification of both effectors and pathogen-modulated host-functions is currently a major goal to unravel molecular basis of plant-microbe interactions [6]. To date, most fungal effectors identified are lineage-specific small-secreted proteins (SSP) of unknown function and hundreds of such SSP coding-genes (i.e. candidate effectors) have been identified in fungal genomes [7]–[11]. Host functions targeted by bacterial effectors are mostly restricted to the plant immune system signaling network, vesicle trafficking, protein degradation systems and transcription of specific transporters [12]. Only a handful of eukaryote effector targets are currently known but the larger effector candidate repertoires compared with those of prokaryotes suggests specialized host manipulations likely linked to nutrient acquisition strategies, infection structures (e.g. haustoria) formation and host reprogramming [13]–[15].

Genome sequences of *Populus trichocarpa* and *Melampsora larici-populina* are available, making the poplar-poplar rust interaction an emerging model for post-genomic studies of tree immunity and fungal biotrophy [16]–[17]. Several genome-wide analyses uncovered the huge expansion and diversification of defense- or virulence-related gene families in poplar and the poplar rust fungus, respectively (for a recent review, see [18]). Several transcriptomic studies identified defense response genes in poplar upon infection by *Melampsora* spp. between 2 and 10 days after inoculation during compatible or incompatible interactions (for a review, see [19]). *M. larici-populina* transcriptome analyses were also conducted during a compatible time-course infection as well as in specific infection structures captured by laser microdissection at late sporulating stages [11], [20]–[21]. A major output of these studies is the timely coordinated and structure-specific expression of hundreds of genes encoding SSPs all along the infection process, suggesting an early and sustained host manipulation [18]. Contrasting with the flow of data gained on both the host and the pathogen transcriptomes at late stages of infection, our knowledge of the first two days of infection remains limited, whereas they encompass a crucial step in setting the outcome of the interaction.

Next-generation sequencing technologies have been an important breakthrough for sensitive, quantitative, annotation-independent and high-throughput transcriptome analyses referred to as RNA-Seq [22]–[24]. Recent works reported the use of RNA-Seq (Illumina or 454-pyrosequencing) to get insights into the transcriptomes of plant-microbe associations [25]–[27]. Using original or phylogenetically related reference genomes, the authors were able to discriminate microbial and host transcripts. In the poplar-poplar rust pathosystem, the availability of both well-annotated genome sequences would greatly facilitate this kind of approach.

In the present study, we used 454-pyrosequencing to analyse the transcriptome of compatible and incompatible poplar-poplar rust interactions within the first two days of infection. Methodological aspects such as discrimination of reads origin (poplar vs. poplar rust) and poplar RNA-Seq data validation by oligoarrays were considered. This work identified five early-expressed candidate effectors from *M. larici-populina* as well as an early-induced sulfate transporter from poplar (*PtSultr3;5*). These data complete our knowledge of the poplar-poplar rust interaction and will help prioritizing functional studies in this pathosystem.

## Results and Discussion

### 454-pyrosequencing Transcriptome of Poplar Leaves Infected by *M. larici-populina*

A time-course infection of poplar leaf disks inoculated with either the virulent strain 98AG31 (compatible interaction) or the avirulent strain 93ID6 (incompatible interaction) of *M. larici-populina* has been established and tissues were harvested at 18, 21 and 24 hours post-inoculation (hpi) for the incompatible interaction (I_18_-I_21_-I_24_) and 18, 24 and 48 hpi for the compatible interaction (C_18_-C_24_-C_48_) (see Fig. S1 for a summary of all experiments and analyses presented in this study). *M. larici-populina* haustoria are formed in both compatible and incompatible interactions as soon as 17 hpi [28], and the first poplar defense responses are observed only in incompatible interactions at 48 hpi [29]. Studies conducted on the flax rust and the bean rust model systems showed that haustoria are key structures for rust fungi to secrete effectors into their host [30]–[33]. Thus, our experiment was designed to identify early-expressed putative rust effectors (i.e. after haustoria formation, between 18 and 24 hpi) as well as early-regulated poplar genes not previously reported. A later time-point in the compatible interaction (C_48_) was included to define the dynamic of SSP expression and to detect modulation in the host transcriptome. For each time-point, cDNA were sequenced by 454-pyrosequencing (GS-FLX titanium), generating a total of 841,301 high-quality nucleotide reads (∼304 Mb) with an average length of 356 bp (Table 1, Fig. S2). For the six conditions, we independently assembled reads into contigs. As presented in Table 1, a total of 713,505 reads were assembled into 90,398 contigs. The average contig size is 659 pb whereas the average number of read per contigs is around 8 (Fig. S2). Homology searches against both *P. trichocarpa* and *M. larici-populina* genome sequences attributed more than 97% of the assembled reads (694,138) to poplar (693,489) or the poplar rust (649) genes (Table 1). This approach will hereafter be referred to as ‘contig-blast method’. We used a second method for read processing, which consisted in the mapping of all the reads onto the reference genomes and current available annotations after reassembly using the Program to Assemble Spliced Alignments (PASA; [34]) (herein called ‘read-mapping method’). This method identified 616,879 and 359 reads corresponding to *P. trichocarpa* and *M. larici-populina* genes, respectively, which is comparable to the numbers gained by the contig-blast method (Table 1). Moreover, expression levels of poplar transcripts (i.e. number of reads per transcripts) estimated by both approaches correlated well, indicating that these methods provide similar outputs (Fig. S3). The data processed through the read-mapping method were more stringent and thus retained for the quantitative analysis of poplar transcript levels, whereas the contig-blast method was retained for the analysis of *M. larici-populina* transcripts.

**Table 1:** Summary of 454-pyrosequencing transcriptome data from poplar leaves infected by *M. larici-populina.*

	I_18_	I_21_	I_24_	C_18_	C_24_	C_48_	Total
Total reads	113,884	155,204	99,033	142,092	146,231	184,856	841,301
Contig-blast method							
Assembled reads	96,078	136,186	81,330	118,783	122,352	158,776	713,505
Contigs	12,938	16,611	12,434	14,391	14,999	19,025	90,398
Poplar reads	93,107	132,086	78,396	116,052	119,217	154,631	693,489
*M.larici-populina* reads	129	43	57	36	37	347	649
Expressed *M. larici-populina* transcripts	31	26	26	27	33	265	361
Read-mapping method							
Poplar reads^c^	82,865	115,272	71,596	105,341	105,933	135,872	616,879
Expressed poplar transcripts (>1 read)	12,518	14,090	12,698	14,050	14,374	15,868	21,311 (6,755)^a^
*M. larici-populina* reads	17	13	10	12	26	281	359
Expressed *M. larici-populina* reads	15	13	9	12	24	226	280
Highly expressed (HE) poplar transcripts (≥10 reads)	2,064	2,086	2,256	2,058	2,039	2,030	978 (1,909)^b^

a Number of poplar transcripts expressed in all conditions.

b Number of poplar transcripts with an average number of 10 reads (i.e. a total of 60 reads in the 6 conditions).

c Normalised number of reads per conditions used for quantitative analysis is 102,813 (Table S1).

### *M. larici-populina* Early-expressed Transcripts and Candidate Effectors

A total of 649 reads were aligned to 361 *M. larici-populina* genes by the contig-blast method, whereas 359 reads were mapped to 280 *M. larici-populina* genes by the read-mapping method (Table 1). In total, 97% of the genes identified by the read-mapping method were also retrieved by the contig-blast method. The lower number of genes identified by the read-mapping method are due to intrinsic differences in assembly parameters. The C_48_ condition itself accounted for almost half of the reads whatever method used, which is in accordance with the fungal growth previously reported at this stage of the infection [29], [35]. The low number of reads does not allow proper quantitative comparison of fungal transcripts abundance between time-points. Thus, we focused our attention on the 40 genes cumulating a total of three reads or more with any of the two methods (Table 2). Among those, a total of 21 genes encode secreted proteins, of which 19 correspond to small secreted proteins of less than 300 amino acids, previously reported as putative candidate effectors of the poplar rust fungus [11]. Despite a very low level of fungal transcripts detected in infected leaf samples, the high number of transcripts encoding putative effectors is striking and in accordance with the levels reported in purified haustoria of other fungal biotroph [32], [36] or in *M. larici-populina* biotrophy-related structures microdissected from infected poplar tissues [20]. Noteworthy, *Mlp*-124067 has been reported among the most highly expressed fungal transcript at 24 hpi during the compatible poplar-poplar rust interaction [21]. The most expressed transcript (*Mlp*-94736, 92 reads) encodes a SSP of 158 amino acids with no functional annotation and only a few homologs in databases (Table 2). Strinkingly, expression of the transcript is mostly explained by the I_18_ time-point that cumulates 86 reads. *Mlp*-94736 belongs to a small cluster of genes within 40 kb on the scaffold 72 of which 4 genes (*Mlp*-94735, *Mlp*-94736, *Mlp*-94740 and *Mlp*-94741) were expressed in the dataset. The second most expressed transcript (*Mlp*-95625, 36 reads) encodes a SSP of 89 amino acids that possesses a short inositol phosphatase domain (conserved domain cd01640). Recent studies have reported that entry of oomycete and fungal effectors into plant cells is mediated by binding to phosphatidylinositol phosphates (PIP) present on the extracellular face of the host plasmalemma [37]–[39]. The early expression of a secreted inositol phosphatase by the poplar rust fungus is puzzling and it is tempting to speculate a role related to the PIP-binding of effectors. Several genes showing homology to ribosomal proteins and elongation factors were also identified (Table 2), which is in accordance with the profile of haustoria expressed transcripts of the obligate biotrophic fungus *Golovinomyces orontii* [36]. Noticeable differences in reads numbers could be observed between the two read assignment methods, particularly for the genes cumulating the largest numbers of reads with the contig-blast method (Table 2). We manually validated the alignment of these contigs onto the *M. larici-populina* genome and their absence with the second method is due to differences in alignment parameters.

**Table 2:** List of selected *M. larici-populina* (Mlp) genes expressed at early stages of poplar leaf infection and cumulating three reads for all time-points assessed in the study with any of the read assignment method.

*Mlp* Protein-ID^a^	reads no. with *contig-blast* method	reads no. with *read-mapping* method	Annotation notes^b^	SSP^c^	Expression notes^d^
13047	5	3	predicted secreted protein of 336 amino acids, no conserved domain	–	NA
33161	3	3	elongation factor elF1b, predicted as secreted	yes	NA
33293	11	0	NADH:ubiquinone oxydoreductase	–	Expressed *in planta* at 24, 48, 96 and 168 hpi and in resting/germinating urediniospores; peaks of expression at 96 and 168 hpi and in urediniospores
36702	3	3	pyruvate dehydrogenase	–	Strongly expressed *in planta* at 48, 96 and 168 hpi and in resting/germinating urediniospores and expressed at 24 hpi; peak of expression in resting urediniospores
42122	5	5	ribosomal protein S2	–	Expressed *in planta* at 24, 48, 96 and 168 hpi and in resting urediniospores; peaks of expression at 48 and 96 hpi and in resting urediniospores
42605	3	3	aspartate/other aminotransferase	–	Expressed *in planta* at 48, 96 and 168 hpi and in resting/germinating urediniospores; peaks of expression in resting urediniospores
53922	3	3	40S ribosomal protein 26S	–	Expressed *in planta* at 24, 48, 96 and 168 hpi and in resting/germinating urediniospores; peak of expression at 96 hpi
70937	4	0	predicted small secreted protein of 286 amino acids with conserved rare lipoprotein A domain	yes	Expressed *in planta* at 168 hpi and in resting/germinating urediniospores; peak of expression in urediniospores
71305	3	0	uncharacterized conserved protein	–	Strongly expressed in resting/germinating urediniospores and expressed *in planta* at 96 and 168 hpi; peak of expression in germinating urediniospores
71396	4	0	predicted small secreted protein of 134 amino acids, no conserved domain	yes	Expressed *in planta* at 48, 96 and 168 hpi and in resting/germinating urediniospores; peaks of expression in germinating urediniospores
74948	3	0	elongation factor elF2	–	Strongly expressed *in planta* at 24, 48, 96 and 168 hpi and in resting/germinating urediniospores; peak of expression in resting urediniospores
84328	6	3	predicted small secreted protein of 147 amino acids, no conserved domain	yes	Expressed *in planta* at 24, 48, 96 and 168 hpi and in germinating urediniospores; peak of expression at 96 hpi
85484	4	4	predicted small secreted protein of 256 amino acids, no conserved domain	yes	Strongly expressed only *in planta* at 24, 48, 96 and 168 hpi; peaks of expression at 48 and 96 hpi
87680	5	0	predicted secreted protein of 536 amino acids with conserved ubiquitin domain	–	NA
88357	3	3	predicted small secreted protein of 163 amino acids, no conserved domain	yes	Strongly expressed *in planta* at 24, 48 and 96 hpi and expressed at 168 hpi; peaks of expression at 48 and 96 hpi
88509	4	0	hypothetical protein of 1455 amino acids, with conserved signaling SWIFT domain	–	Strongly expressed in resting/germinating urediniospores and expressed in planta at 24, 48, 96 and 168 hpi; peak of expression in germinating urediniospores
90053	12	0	hypothetical protein of 161 amino acids with zinc-finger domain	–	ND
91962	4	4	elongation factor elF5A	–	Strongly expressed *in planta* at 24, 48, 96 and 168 hpi and in resting/germinating urediniospores; peak of expression at 168 hpi and in urediniospores
92704	5	0	hypothetical protein of 195 amino acids, no conserved domain	–	Strongly expressed *in planta* at 24, 48, 96 and 168 hpi and in resting/germinating urediniospores; peaks of expression at 24 and 48 hpi
92712	3	0	hypothetical protein of 131 amino acids, no conserved domain	–	Strongly expressed *in planta* at 24, 48 and 96 hpi and in resting/germinating urediniospores and expressed at 168 hpi; peak of expression at 24 hpi
93158	4	4	60S ribosomal protein L10a	–	Strongly expressed *in planta* at 96 hpi and in resting urediniospores and expressed at 24, 48 and 168 hpi; peak of expression in urediniospores
93408	1	3	predicted small secreted protein of 147 amino acids, no conserved domain	yes	Strongly expressed *in planta* at 24, 48 and 96 hpi and also expressed at 168 hpi and in resting/germinating urediniospores; peaks of expression at 24 and 48 hpi
94735	9	0	predicted small secreted protein of 95 amino acids, no conserved domain	yes	Strongly expressed *in planta* at 24, 48, 96 and 168 hpi and in resting/germinating urediniospores; peaks of expression at 24 hpi
94736	92	0	predicted small secreted protein of 158 amino acids, no conserved domain	yes	NA
94740	8	0	hypothetical protein of 176 amino acids, no conserved domain	–	NA
94741	5	0	hypothetical protein of 77 amino acids, predicted transmembrane domain	–	NA
95026	3	0	hypothetical protein of 210 amino acids, no conserved domain	–	NA
95625	36	0	predicted small secreted protein of 89 amino acids with inositol polyphosphate-1-phosphatase domain	yes	NA
96323	4	0	hypothetical protein of 537 amino acids, HMG-box containing domain	–	ND
103016	3	3	predicted small secreted protein of 139 amino acids, no conserved domain	yes	Strongly expressed in planta at 24, 48 and 96 hpi and expressed at 168 hpi and in resting/germinating urediniospores; peaks of expression at 24, 48 and 96 hpi
105045	3	3	predicted small secreted protein of 124 amino acids, no conserved domain	yes	Expressed *in planta* at 24, 48, 96 and 168 hpi and slightly in germinating urediniospores; peak of expression at 96 hpi
110164	3	0	hypothetical protein of 211 amino acids, zinc-finger containing domains	–	ND
118176	3	0	hypothetical protein of 1096 amino acids, HMG-box containing domain	–	Expressed in planta at 168 hpi and in resting/germinating urediniospores; peak of expression in germinating urediniospores
123227	3	3	predicted small secreted protein of 124 amino acids, no conserved domain (SSP15)	yes	Strongly expressed only *in planta* at 168 hpi and slightly expressed at 24, 48 and 96 hpi; peak of expression at 168 hpi
124018	3	3	predicted small secreted protein of 156 amino acids, no conserved domain	yes	Strongly expressed *in planta* at 24, 48, 96 and 168 hpi and slightly expressed in resting/germinating urediniospores; peaks of expression at 48 and 168 hpi
124067	5	4	predicted small secreted protein of 285 amino acids with homology to *Uromyces* Infp differentiation protein	yes	Strongly expressed *in planta* at 24, 48, 96 and 168 hpi and in resting/germinating urediniospores; peak of expression at 96 hpi
124290	1	4	predicted small secreted protein of 131 amino acids, no conserved domain	yes	Strongly expressed *in planta* at 24, 48 and 96 hpi and expressed at 168 hpi and in resting/germinating urediniospores; peaks of expression at 48 hpi
124371	3	2	predicted small secreted protein of 89 amino acids, no conserved domain	yes	NA
124458	3	3	conserved small secreted protein of 270 amino acids, with CFEM domain	yes	Strongly expressed *in planta* at 24, 48, 96 and 168 hpi and expressed in resting/germinating urediniospores; peaks of expression at 48 and 96 hpi
124534	3	3	predicted small secreted protein of 70 amino acids, no conserved domain	yes	NA

a Protein ID number of corresponding best gene model in the *M. larici-populina* genome sequence (JGI; http://genome.jgi-psf.org/programs/fungi/index.jsf);

b Based on annotation details available on the JGI website and homology searches against the non-redundant database and the conserved domain database at the NCBI;

c predicted small secreted protein (SSP; ≤300 amino acids);

d Based on expression data reported in Duplessis et al. 2011b [**21**];

NA, not available on oligoarray;

ND, not detected on oligoarray.

Expression of *M. larici-populina* transcripts during poplar leaf colonization (compatible interaction) and urediniospore germination was previously assessed by the mean of Genome oligoarrays [21]. Among the set of 40 fungal genes, 30 were represented on the oligoarray, of which 27 are expressed in the situations tested and particularly, 24 are expressed at 24 and 48 hpi, which provides a nice support for the transcripts detected by the 454-pyrosequencing approach. Overall, the low number of fungal transcripts detected in our study reflects the limited quantity of fungal structures in early-infected leaves [29], [35]. In comparison, Fernandez and collaborators [27] identified almost one fungal for two host transcripts in the coffee-coffee rust fungus interaction, however this study targeted a late stage of infection when the fungus has completely invaded host tissues. Although providing valuable insights into early-expressed fungal transcripts, our analysis remains scale-limited and argues for the use of other NGS technologies such as Illumina for a deeper sampling of the pathogen transcriptome.

### 454-pyrosequencing does not Saturate the Poplar Transcriptome

A total of 616,879 reads were mapped onto 21,311 poplar transcripts (i.e. predicted protein coding genes in poplar genome), considered as expressed when at least one read was mapped (Table 1). Among these 21,311 transcripts only 6,755 were expressed in all conditions. The average and median of total reads number per expressed transcript are 28.95 and 8 respectively, ranging from 1 to 6,513 (Fig. S4A). Saturation curves indicate that 52±5 new expressed transcripts are detected for each 1000 supplemental reads after 100,000 (Fig. S4B). This demonstrates that the poplar transcriptome is not saturated and consequently unsuitable for whole-genome quantitative expression analysis. As specified recently by Malone and Oliver [40], an important concern in RNA-Seq experiments is the depth of sequencing required to efficiently sample the transcriptome. By using Illumina sequencing, Graveley and collaborators [41] reported that even 50 millions of mapped reads were not enough to fully saturate the fly-head transcriptome. Despite huge progresses in sequencing, it seems that full saturation of transcriptomes still cannot be considered as an easy purpose.

In order to isolate a subset of transcript with a sufficient coverage to enable quantitative analysis of expression, we dramatically reduced our set from 21,311 to 1,909 highly expressed (HE) transcripts, with a minimal arbitrary cut-off of 60 total reads per HE transcript (Table 1, Fig. S4).

### Poplar RNA-Seq Expression Levels are Validated by Oligoarrays and RT-qPCR

**Figure 1:**
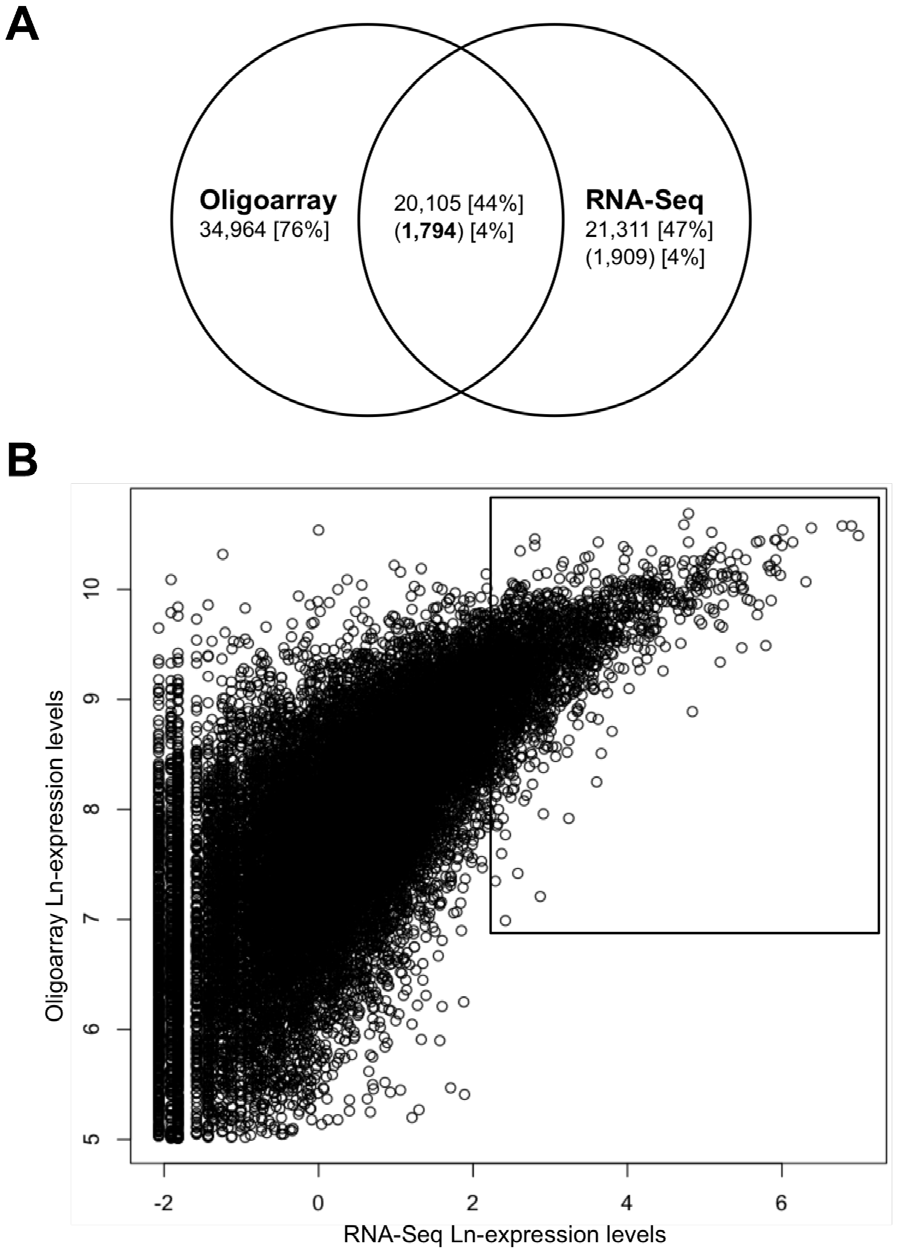
Ln-transformed expression levels of poplar transcripts. (A) Venn-diagram of expressed poplar transcripts measured by whole-genome oligoarray and RNA-Seq. Numbers in brackets correspond to the percentage of the coding genome; numbers in parentheses correspond to the highly expressed (HE) transcripts and numbers in parentheses and in bold correspond to HE transcripts validated by oligoarrays (i.e. VHE transcripts). (B) RNA-Seq and oligoarrays average expression levels correlation. The black rectangle indicates the area containing the 1,794 VHE transcripts discussed in the text.

In order to validate poplar RNA-Seq data, we performed whole-genome poplar oligoarrays and compared expression values gained by both methods. The DNA-chip approach detected the expression of 34,964 transcripts (76% of the coding genome) of which 31,644 were expressed in all conditions (Fig. 1a). Compared with the 21,311 expressed transcripts revealed by RNA-Seq, oligoarrays clearly demonstrate that the RNA-Seq approach was not deep enough to cover the entire poplar transcriptome. Oligoarrays confirmed the expression of 20,105 of the 21,311 transcripts detected as expressed by RNA-Seq (Fig. 1a). Among the 1,206 unconfirmed transcripts, 440 were detected below the oligoarrays background threshold whereas 766 were not spotted on the chip. Otherwise, 1,794 of the 1,909 HE transcripts were detected as highly-expressed on oligoarrays and are hereafter referred to as validated highly expressed (VHE) transcripts. Among the 115 non-validated HE transcripts, only 5 were detected below the threshold whereas 110 were not included in the chip design. The expression levels of the 1,794 VHE transcripts assessed by oligoarray and RNA-Seq showed a good correlation (r^2^ = 0.37) that increases with expression level (Fig. 1b). To further validate the RNA-Seq expression profiles, we tested 12 selected VHE transcripts by RT-qPCR. The expression profiles were in agreement with those obtained with the two other methods (Fig. S5). Otherwise, it is noteworthy to mention that 20% of the most highly expressed transcripts on oligoarrays were not supported by RNA-Seq, revealing a unidirectional and non-negligeable technical biase between these approaches discussed in Text S1 (Fig. S6).

### Transcriptome of Poplar Leaves: Overall Stability at Early Stages of Infection

**Figure 2:**
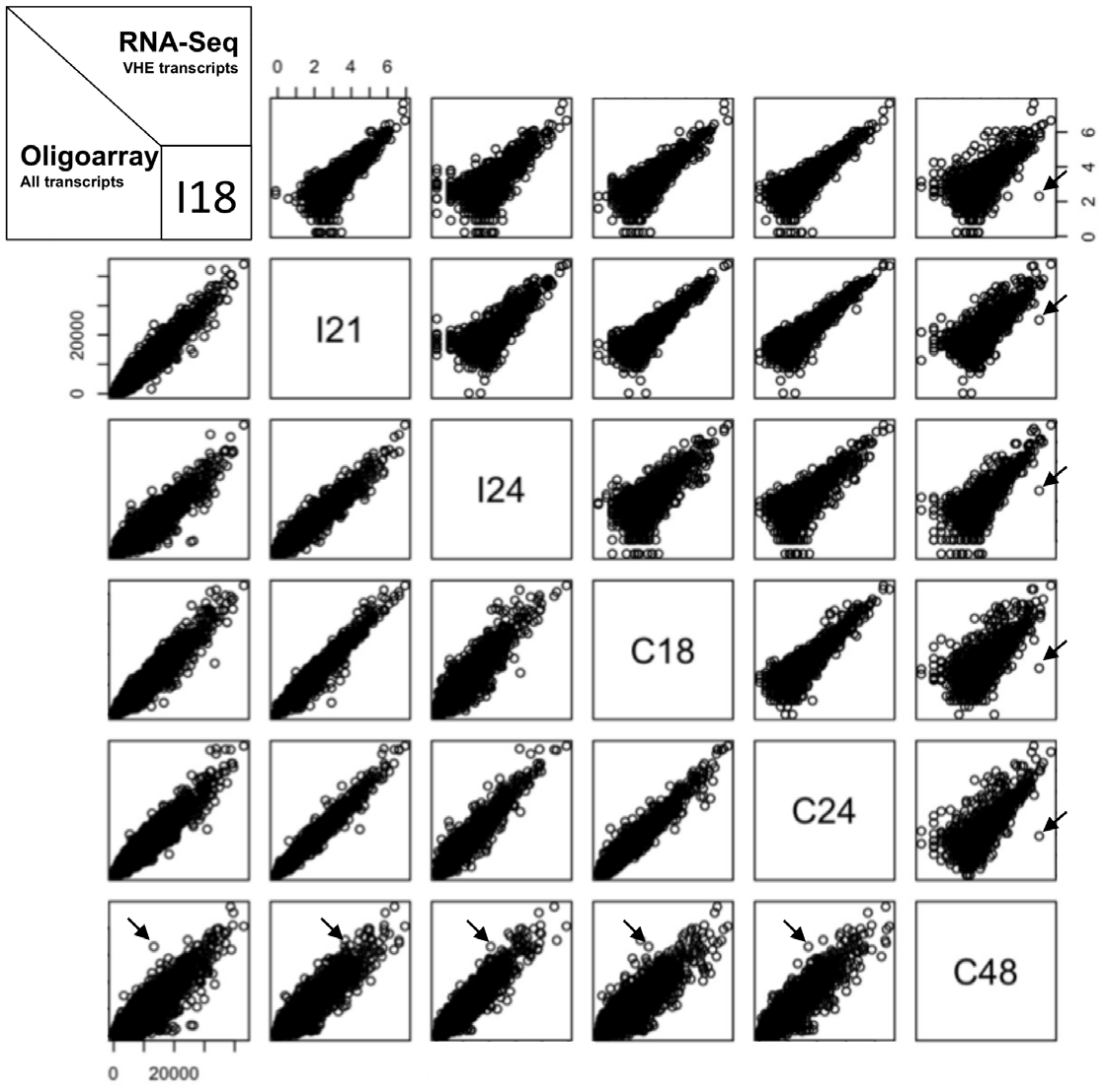
RNA-Seq and oligoarray two-by-two expression levels correlations along time-course infection of poplar leaves by *M. larici-populina*. Ln-transformed expression levels of the 1,794 validated highly expressed (VHE) transcripts are plotted for RNA-Seq on the up-right, and expression levels of the 34,964 transcripts detected on oligoarrays are plotted on the bottom-left. Arrows indicate values corresponding to the sulfate transporter PtSultr3;5 (Poptr_0006s16150) transcript discussed in the text.

In order to identify early-regulated transcripts in the poplar transcriptome, expression levels were compared pair-by-pair between the 6 different conditions tested (Fig. 2). Among the VHE transcripts, 1,793 showed overall stable RNA-Seq expression levels, with average relative standard deviation percentage (%RSD, see methods) values of 35, ranging from 2 to 101 (Fig. S7a). The only highly variable transcript with a %RSD of 186 is a predicted sulfate transporter homologous to *Arabidopsis thaliana* Sultr3;5 (AtSultr3;5) [42] and hybrid poplar *Populus tremula* x *Populus alba* Sultr3;5 (PtaSultr3;5) [43] and thus was herein named *P. trichocarpa* Sultr3;5 (PtSultr3;5). The overall stability of VHE transcript expression levels was confirmed by oligoarrays, which present %RSD values from 1 to 62 (Fig. S7b). Moreover, expression levels of the 34,964 oligoarray-expressed transcripts were equally stable, with only 18 transcripts with %RSD above 100, which all correspond to low and barely expressed genes (Fig. 2, Table S1). Functional classification of the 1,794 VHE transcripts as well as detailed analysis of the stress responsive phenylpropanoid pathway further confirmed the absence of defense responses (Text S2, Fig. S8 and S9). Hence, except *PtSultr3;5,* the leaf transcriptome remains extremely stable for the time-points investigated.

Many host transcriptomes have been reported to be highly dynamic during the first events of plant-microbe interactions [44]–[45], as highlighed recently in soybean leaves as early as 12 hours after inoculation by the rust fungus *Phakospora pachyrhizi* [46]. Nevertheless, in the poplar-poplar rust system, typical defense-response genes encoding pathogenesis-related (PR) proteins such as PR1 or PR5, glutathion S-transferase 18 (GST18) or the rust-induced secreted protein (RISP) are not regulated and present a basal expression at early stages of leaf infection (Table S1, [29], [47]). Indeed, the first defense responses are reported at 48 hpi in the incompatible interaction, concomitant with an arrest of *M. larici-populina* avirulent strain progression in leaves [28]–[29]. Considering the global stability of the poplar trancriptome and the fact that sulfate transporters are not reported as typical defense-related genes in the literature, we conclude that no defense responses could be detected in early rust-infected poplar leaves.

### A Poplar Sulfate Transporter is Specifically Induced by the Rust Fungus

**Figure 3:**
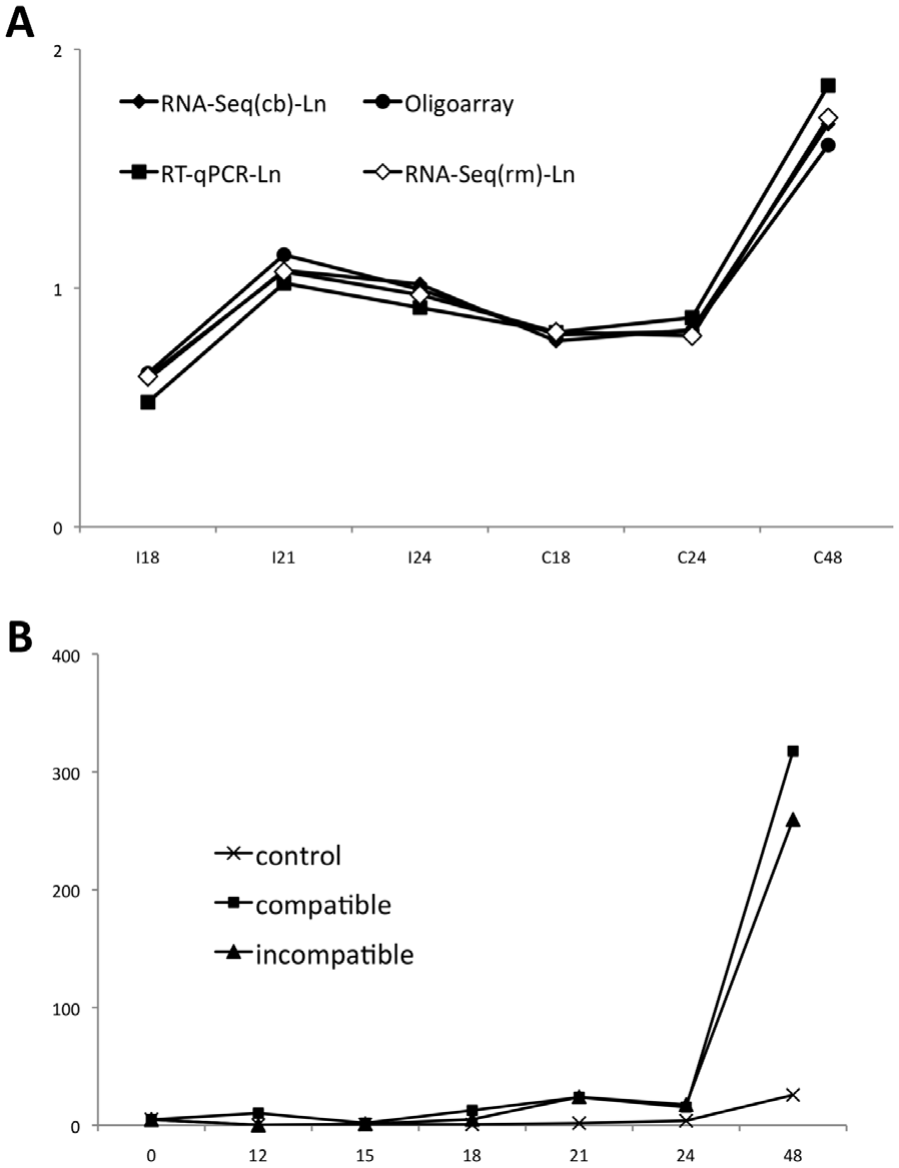
Expression profile of *PtSultr3;5*. (A) Expression pattern of *PtSultr3;5* (Poptr_0006s16150) assessed by oligoarray, RT-qPCR and RNA-Seq. RNA-Seq and RT-qPCR values are Ln-transformed. For RNA-Seq, both results from the contig-blast (cb) and the read-mapping (rm) methods are presented. Values are in arbitrary units. (B) Detailed RT-qPCR profile of *PtSultr3;5* between 0 and 48 hours post-inoculation (hpi). Expression values are normalized to the ubiquitin reference gene expression (see methods).

The sulfate transporter *PtSultr3;5* displayed the highest %RSD among all VHE transcripts (Fig. S7). Interestingly, the variability observed is explained by the condition C_48_ (515 reads) with an almost 20-fold up-regulation compared with the other five conditions that remain stable (26±15 reads) (Table S1). The different methods used in this study to quantify gene expression provided highly similar profiles for this gene, validating its effective regulation (Fig. 3a). Moreover, complementary infection time-points included in the RT-qPCR assay, as well as comparison to mock-inoculated control leaves, provided a more accurate expression pattern (Fig. 3b). A strong induction of *PtSultr3;5* was observed at 48 hpi in both compatible and incompatible poplar-polar rust interactions compared with all other conditions that showed an overall stable expression. Thus, *PtSultr3;5* presents a fungal-specific strain-independent induction in poplar leaves. Since we did not detect neither defense responses nor large-scale transcriptome regulations, and considering the specificity and amplitude of the induction of *PtSultr3;5*, we hypothesized that this specific up-regulation could result from a pathogen manipulation.

PtSultr3;5 is the ortholog of AtSultr3;5 (At5g19600) that belongs to the family of low-affinity sulfate transporter and is localized at plasma membranes [48]. Interestingly, PtSultr3;5 is also the ortholog of the *Lotus japonicus* symbiotic sulfate transporter 1 (SST1) with 57% of identity (Fig. S10). In *L. japonicus* intracellular rhizobia symbiotic interactions, SST1 is located onto the symbiosome membrane, the interface for host-symbiont nutrient exchanges [49]. Moreover, Krusell and colleagues [50] demonstrated that *sst1* expression is nodule-specific and that a mutant lacking *sst1* was defective for symbiosis. The authors concluded that SST1 is essential for symbiosis, likely by translocating sulfate from the host cell to the symbiont. Interestingly, Felten and collaborators [51] reported that *PtSultr3;5* is one of the 15 most induced transcripts in poplar roots during early interaction with the symbiotic fungus *Laccaria bicolor*. In these experiments, the host and its symbiont where not in contact, suggesting that indirect and diffusible signals were directly or indirectly responsible of host-transcriptome reprogramming.

No obvious link has been established so far between host sulfur metabolism and mechanisms of pathogenicity in biotrophs. However, in a recent groundbreaking study, Mukhtar and collaborators [52] showed that multiple effectors from divergent biotroph pathogens target response to low sulfur proteins 1 and 2 (LSU1 and LSU2). As mentioned by the authors, the identification of sulfur-related proteins as conserved targets of effectors is puzzling since one of the hallmarks of biotrophy is the loss of key genes necessary to assimilate sulfur. Indeed, this has been reported for several biotroph pathogens belonging to distinct phyla such as the oomycete *Hyaloperonospora arabidopsidis*, the ascomycete *Blumeria gaminis* and the basidiomycete *Puccinia graminis* f. sp. *tritici* [9], [17], [53]–[54]. In the case of *M. larici-populina*, genes required to perform the primary sulfate assimilation were nevertheless identified, although a transketolase domain of the beta-subunit of the sulfide reductase is missing [17]. Interestingly, 2 out of 3 *M. larici-populina* sulfate transporter transcripts are expressed during poplar leaf infection and in urediniospores, however sulfate metabolism transcripts are barely detected [21].

It is tempting to speculate that *M. larici-populina* could perform a targeted-manipulation of poplar physiology by inducing *PtSultr3;5* to derivate sulfate via a host-encoded mechanism. Such a manipulation of host transporter expression by pathogens has been described in interactions between *Xanthomonas* spp. and rice or pepper, where host genes encoding sugar transporters are specifically and necessarily induced by transcription activator-like (TAL) bacterial effectors to settle compatibility [55]–[57]. Interestingly, fungal pathogens also induce sugar exporters during plant infection [57] and although effectors have been brought up, the molecular mechanism underpining this phenomenon remains to be determined [58]. To our knowledge, no TAL effectors have been reported outside of the bacterial genus *Xanthomonas* and *Ralstonia*. Interestingly, a recent report by Plett and colleagues [14] has shown that a small-secreted effector protein from the symbiotic fungus *L. bicolor* targets the host nucleus to settle compatibility through poplar roots transcriptome modulation. Moreover, in plant-oomycete interactions, the massive delivery of effector proteins into the host nucleus has been observed [15], [59]. Altogether, these studies raise interesting perspectives on the ability of biotrophic eukaryote plant symbionts to perform targeted modulations of host genetic programs.

### Conclusions

In this study, we performed a transcriptome analysis of early stages of poplar leaf infection by the rust fungus *M. larici-populina* both during compatible and incompatible interactions. We covered critical steps of the fungus biotrophic growth in the host, when the first haustorial structures are formed ahead of typical host defense responses. Complementary large-scale expression approaches were used including 454 pyrosequencing and oligoarrays to assess the accuracy of the expression data. At these early stages, only a few fungal transcripts were detected *in planta* indicating that 454-pyrosequencing could not support investigation of fungal function. Nevertheless, early-expressed candidate effectors corresponding to probable rust pathogenicity determinants were detected and will be targeted in future investigations. We report the absence of induction of typical defense response genes in infected poplar leaves before 48 hpi, which is contrasted by a highly induced poplar sulfate transporter. This last observation, in the light of our knowledge of biotrophy, is puzzling and establishes a new correlative link between biotrophic growth and host sulfate. *PtSultr3;5* is also early induced in poplar roots upon indirect interaction with a symbiotic fungus (i.e. pre-symbiotic contact) and could represent a conserved mechanism targeted by symbiotic and pathogenic biotrophs to settle compatibility with poplar. Further functional investigations are now needed to understand the role of PtSultr3;5 and more generally to underpin how plant sulfate transport could play a role in plant-microbe interactions.

## Materials and Methods

### Biological Material, Infection Procedures and Time-course Infection

*Melampsora larici-populina* isolates 98AG31 (pathotype 3-4-7) and 93ID6 (pathotype 3-4), respectively virulent and avirulent on the hybrid *P. trichocarpa* x *P. deltoides* poplar cultivar ‘Beaupré’, were used in this study. Urediniospores multiplication and poplar leaf inoculation procedures were carried out as previously described [29] except that inoculations were performed on 5 cm^2^ leaf disks as reported in other studies [60]–[62]. For time-course infection analyses, leaf disks were harvested at 0, 12, 15, 18, 21, 24 and 48 hpi in compatible (C) and incompatible (I) interactions as well as in mock-inoculated (water agar) condition (T). The 6 following conditions were used for 454-pyrosequencing and oligoarrays: I_18_, I_21_, I_24_, C_18_, C_24_, C_48_ (Fig. S1). All of these conditions were used for RT-qPCR analyses. No biological duplication was performed.

### RNA Extraction and cDNA Preparation

Total RNA extraction and quantity/quality control were performed as previously described [29]. For 454-pyrosequencing and oligoarray experiments, tagged double-strands (ds) cDNA were synthesized with the SMARTer PCR cDNA Synthesis Kit (Clontech, Saint-Germain-en-Laye, France), following manufacturer instructions, with 0.5 µg of starting total RNA and 19 cycles of PCR amplification in 25 µL. PCR products were purified with the Quiaquick PCR Purification Kit (Qiagen, Couteboeuf, France), quantified with a nanodrop 1000 spectrophotometer (Labtech, Palaiseau, France) and their quality was assessed by electrophoretic RNA profiling with an Experion analyzer using the Experion RNA Standard-Sens analysis kit (Bio-Rad, Marnes-la-Coquette, France). For RT-qPCR experiments, cDNA were synthesized using the iScript cDNA Synthesis Kit (Bio-Rad,) with 1 µg of total RNA and following manufacturer instructions.

### 454-pyrosequencing and Oligoarrays

Sequences were generated from 5 µg of full-length cDNA using the 454-GS-FLEX Titanium pyrosequencing technology on a Roche 454 Titanium sequencer (single read strategy) at the CEA-Genoscope (Centre National de Séquençage, Evry, France; http://www.genoscope.cns.fr/spip) following standard procedures recommended by Roche. Two half-plates were used: one for the I_18_, I_21_, I_24_ conditions mixed 1/3-1/3-1/3 together with specific tags; and another one for the C_18_, C_24_, C_48_ conditions mixed 1/3-1/3-1/3 together with specific tags. Raw 454-sequences are available upon request. The interest and use of 454-sequences for complementary annotation of poplar and poplar rust genomes are discussed in Text S3. Whole-genome poplar oligoarrays were generated and hybridized at the NimbleGen facilities (NimbleGen systems, Reykjavik, Iceland) following standard protocols [29], [51]. Microarray probe intensities were quantile normalized across chips, using the Arraystar software (DNAStar, Inc., Madison, Wi, USA). The background level was fixed at 150 (average 3-fold random probe intensities across oligoarray hybridizations). The complete oligoarray expression datasets are available at the NCBI GEO website as series number GSE34802.

### RT-qPCR

Specific primers were designed for targeted ‘Beaupré’ transcripts and RT-qPCR were carried out as previously detailed on technical duplicates [20]. Sequences gained by RNA-Seq were used for primer design in order to avoid polymorphism bias during alleles amplifications of the ‘Beaupré’ hybrid cultivar. Transcript expression was normalized to the poplar ubiquitin reference transcript [29] and calculated using the following equation: Relative Expression  =  [(target gene primer efficiency)^−Ct^]/[(reference gene primer efficiency)^−Ct^], where Ct is the number of PCR amplification cycles necessary for signal detection [63]. Primer sequences are presented in Table S2.

### Bioinformatic Procedures for RNA-Seq Data Analysis

Raw reads were processed to remove SmartER adaptor sequences and reads shorter than 70 pb were discarded. For the contig-blast method, reads were assembled into contigs with the Mimicking Intelligent Read Assembly (MIRA) program [64] and blast homology searches were performed against both *M. larici-populina* (version 1.0, JGI website http://genome.jgi-psf.org/Mellp1/) and *P. trichocarpa* (version 2.2, Phytozome website http://www.phytozome.net/poplar) genome sequences. Contigs were assigned to the reference genomes based on blast scores and e-values, and ambiguous assignments were eventually resolved by searching for plant or fungal homologs in international databases as previously described [27]. All assignments were validated by genome assignments with GenomeThreader [65]. For the read-mapping method, reads were aligned directly on *P. trichocarpa* and *M. larici-populina* genome sequences using Program to Assemble Spliced Alignments (PASA, [34], http://pasa.sourceforge.net/) with default parameters and GMAP as the program to align transcripts and annotated coding sequences available at the *P. trichocarpa* and *M. larici-populina* genomes websites. PASA included *de novo* assembly on the genome considering available annotations and proposed predictions for gene correction, gene creation as well as identification of putative splicing variants (see Text S3). Numbers of reads attributed to annotated genes at the six time-points were retrieved using in-house scripts and reads’ numbers were normalized between the six conditions prior further quantitative comparisons (Table 1). Expression analyses and graphics were done with Excel for Mac (Microsoft) or R version 2.9.2 (http://cran.r-project.org/). For a given transcript, %RSD of the six expression values was calculated according to the following equation: %RSD  =  [(standard deviation/average) x 100]. Sequences were analyzed in Text Wrangler version 2.3 (Bare Bones Software, Inc.) and Tablet version 1.10.05.21 [66]. Alignments were performed with the Multalin online software (http://multalin.toulouse.inra.fr/multalin/). The Functional Catalogue (FunCat) version 2.1 was used for classification of genes with slight modifications within categories [67]. Read and contig sequences as well as PASA outputs (i.e. gene model re-annotation as well as identification of alternative splicing and of allelic polymorphism) are available upon request. Saturation curves were performed with Analytic Rarefaction software version 2.0 (http://www.huntmountainsoftware.com/).

## Supporting Information

Figure S1 **Experimental design and bioinformatic procedure summary.** (A) Experimental design of the time-course infection. All conditions and time-points were used for standard cDNA synthesis and subsequent RT-qPCR, whereas the 6 conditions highlighted in black (I_18_, I_21_, I_24_, C_18_, C_24_, C_48_) were used for hybridization on poplar oligoarrays and for tagged cDNA synthesis and subsequent 454-pyrosequencing as depicted in b. (B) Summary of the bioinformatic procedure and analyses performed in the study.
(TIF)

Figure S2 **Read and contig length distribution.**
(TIF)

Figure S3 **RNA-Seq data analysis methods comparison.** RNA-Seq mean expression levels are Ln-transformed.
(TIF)

Figure S4 **RNA-Seq coverage of the poplar transcriptome.** (A) Distribution of RNA-Seq reads among the 21,311 expressed poplar transcripts. The black rectangle highlights the subset of 1,909 highly expressed (HE) transcripts considered for quantitative analyses. (B) Average saturation curve of the poplar transcriptome by RNA-Seq.
(TIF)

Figure S5 **Correlation between RNA-Seq, oligoarrays and RT-qPCR normalized expression levels for 12 selected validated highly expressed (VHE) transcripts (listed in Table S2).**
(TIF)

Figure S6 **Dot-plot of the 285 most-expressed transcripts on oligoarrays (average expression values above 20,000) and corresponding RNA-Seq expression values.**
(TIF)

Figure S7 **Percentage-relative standard deviation (%RSD) distributions.** (A) %RSD for RNA-Seq expression levels of the 1,794 validated highly expressed (VHE) transcripts. (B) %RSD of oligoarray expression levels of the 1,794 VHE transcripts. Arrows in (A) and (B) indicate values of the fungal-induced sulfate transporter (*PtSultr3;5*, Poptr_0006s16150).
(TIF)

Figure S8 **Functional Catalogue (FunCat) classification of the 1,794 validated highly expressed (VHE) transcripts.** (A) Distribution of FunCat categories. Values represent percentage of the 1,794 VHE transcripts. (B) Average expression levels in FunCat categories. (C) Average percentage-relative standard deviation (%RSD) in FunCat categories.
(TIF)

Figure S9 **Transcriptional levels of the phenylpropanoid pathway transcripts in poplar leaves infected by** ***M. larici-populina*** **.**
(TIF)

Figure S10 **Alignment of selected plant sulfate transporters homologous to PtSultr3;5.** Amino acid sequences of PtSultr3;5 (*Populus trichocarpa*, POPTR_0006s16150.1), Beaupré-9 (contig C48-lrc9, *P. trichocarpa* x *Populus deltoides* ‘Beaupré’), Beaupré-124 (contig C48-lrc-124, *P. trichocarpa* x *P. deltoides* ‘Beaupré’), AtSultr3;5 (*Arabidopsis thaliana*, At5g19600), NCBI ID 117557144 (PtaSultr3;5, *Populus tremula* x *Populus alba*), NCBI ID 255549068 (*Ricinus communis*), NCBI ID 225445290 (*Vitis vinifera*), NCBI ID 45720463 (*Brassica oleracea*), NCBI ID 297812143 (*Arabidopsis lyrata*) and SST1 (*Lotus japonicus*). Asterisks indicate polymorphic residues between ‘Beaupré’ alleles and the double-head arrow marks additional predicted amino acids in the *P. trichocarpa* ‘Nisqually-1’ genome discussed in Test S3.
(TIF)

Table S1 **Summary table of expression, annotation and sequence data used in the study for poplar genes.**
(ZIP)

Table S2 **List of primers used in this study.**
(XLS)

Text S1 **Uncorrelated oligoarray/RNA-Seq transcript expression.**
(DOC)

Text S2 **Functional classification of the 1,794 poplar validated highly expressed (VHE) genes.**
(DOC)

Text S3 **Interest and use of 454-sequences for complementary annotation of poplar and poplar rust genomes.**
(DOC)
