# Supplementary figures and images for: RNA-Seq of Early-Infected Poplar Leaves by the Rust Pathogen Melampsora larici-populina Uncovers PtSultr3;5, a Fungal-Induced Host Sulfate Transporter
Source: PLoS One. 2012 Aug 30;7(8):e44408. doi: 10.1371/journal.pone.0044408 (PMC3431362; doi:10.1371/journal.pone.0044408)

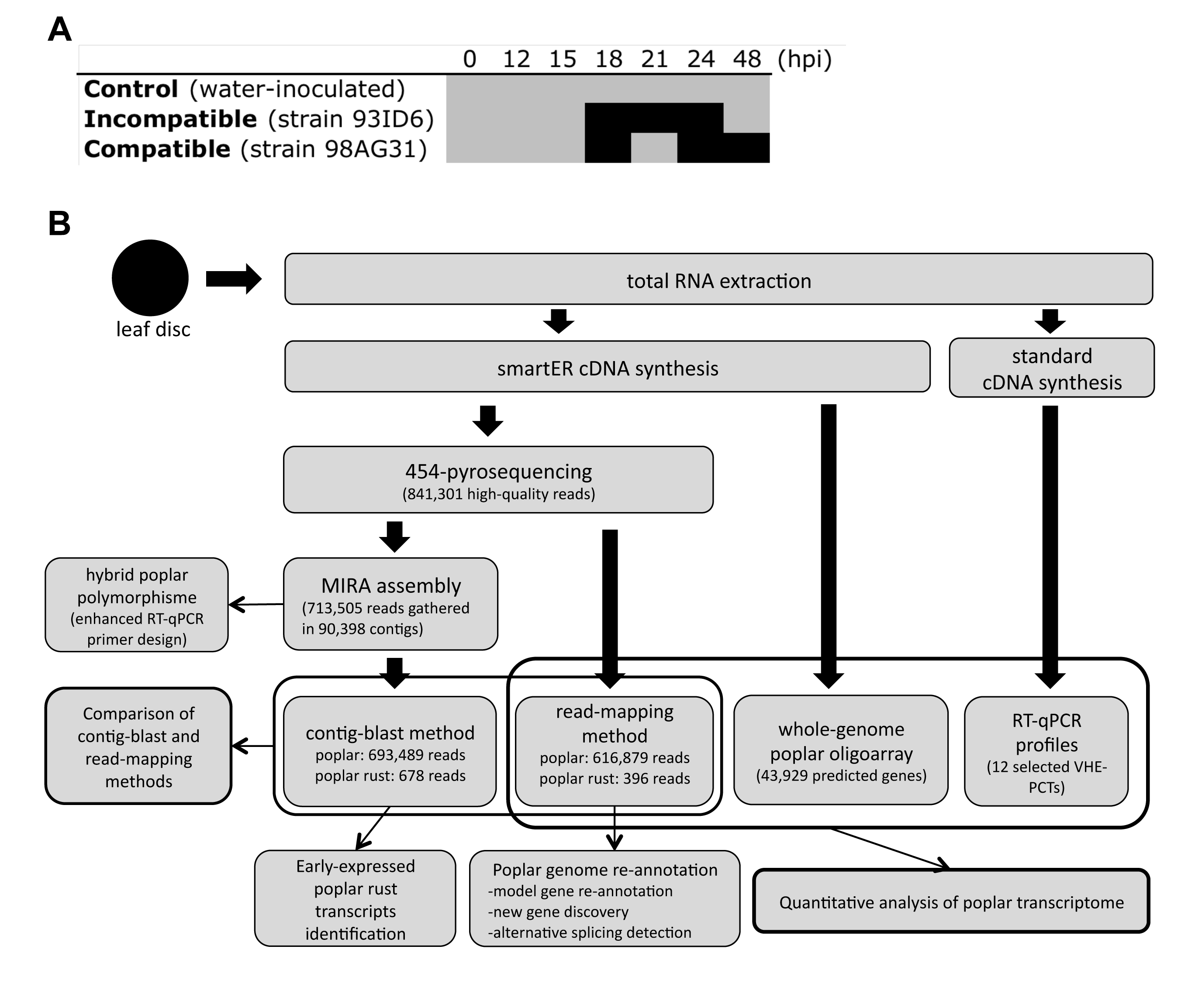

Supplement: Figure S1 — Experimental design and bioinformatic procedure summary. (A) Experimental design of the time-course infection. All conditions and time-points were used for standard cDNA synthesis and subsequent RT-qPCR, whereas the 6 conditions highlighted in black (I18, I21, I24, C18, C24, C48) were used for hybridization on poplar oligoarrays and for tagged cDNA synthesis and subsequent 454-pyrosequencing as depicted in b. (B) Summary of the bioinformatic procedure and analyses performed in the study. (TIF) [file pone.0044408.s001.tif]

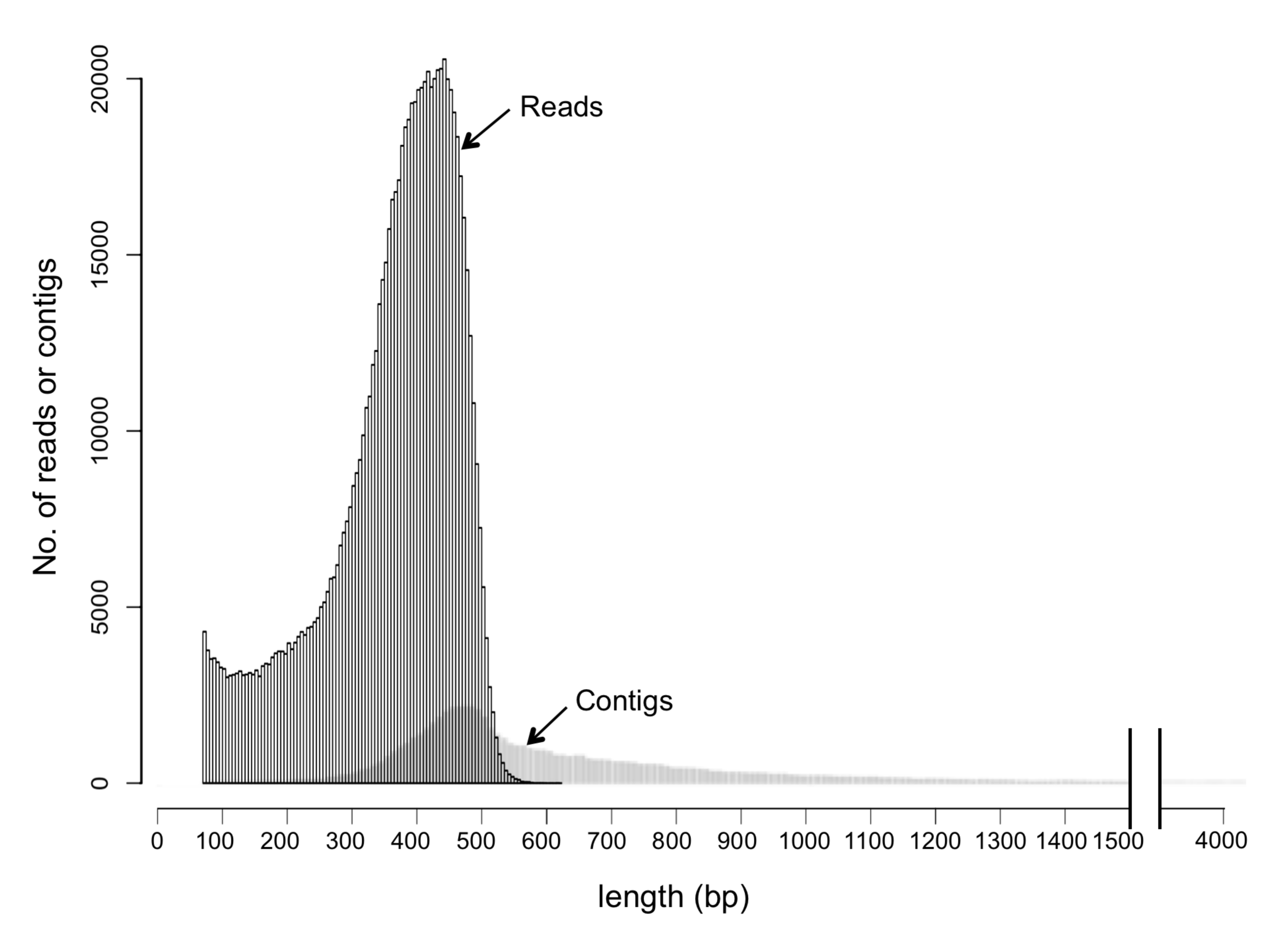

Supplement: Figure S2 — Read and contig length distribution. (TIF) [file pone.0044408.s002.tif]

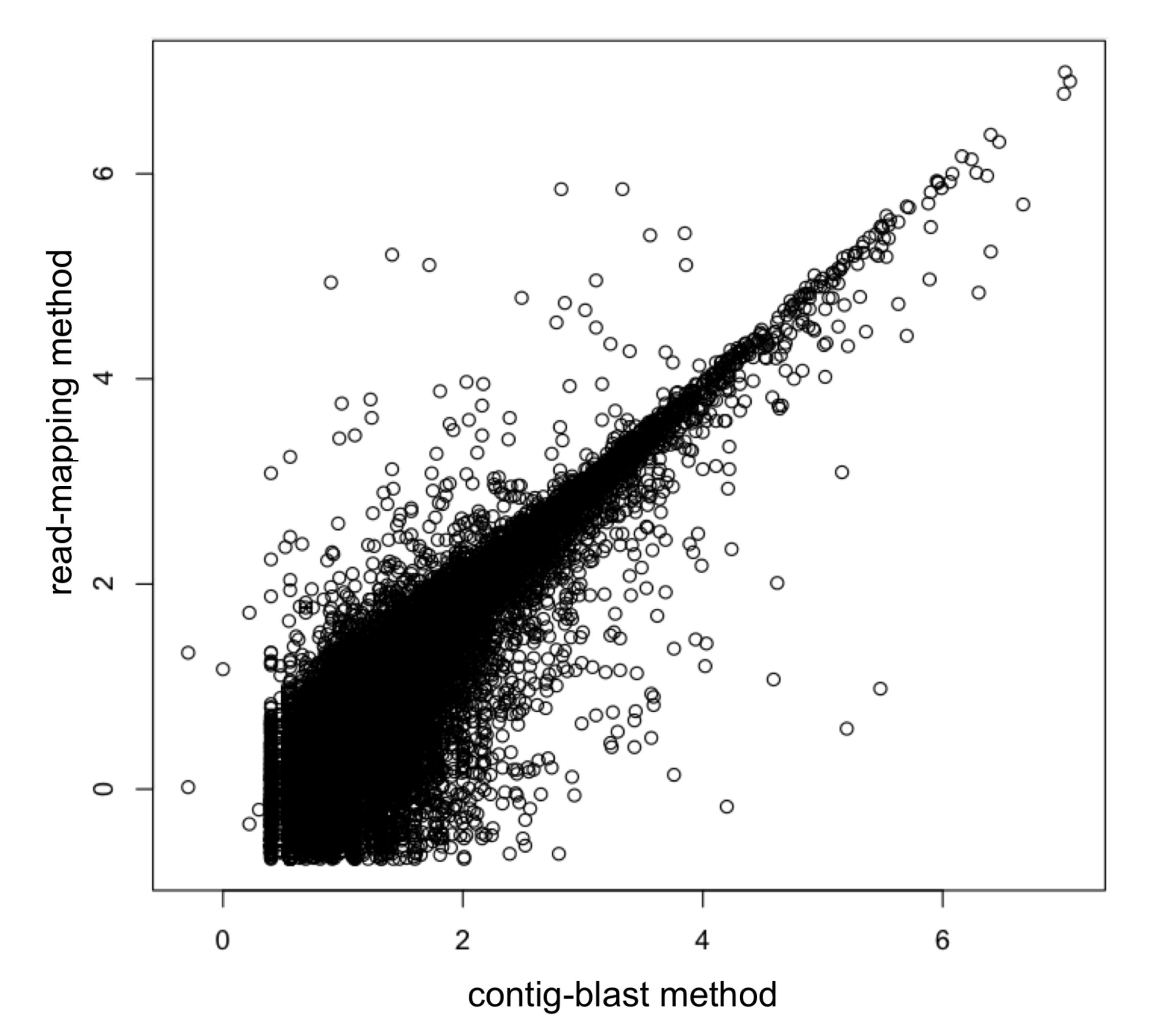

Supplement: Figure S3 — RNA-Seq data analysis methods comparison. RNA-Seq mean expression levels are Ln-transformed. (TIF) [file pone.0044408.s003.tif]

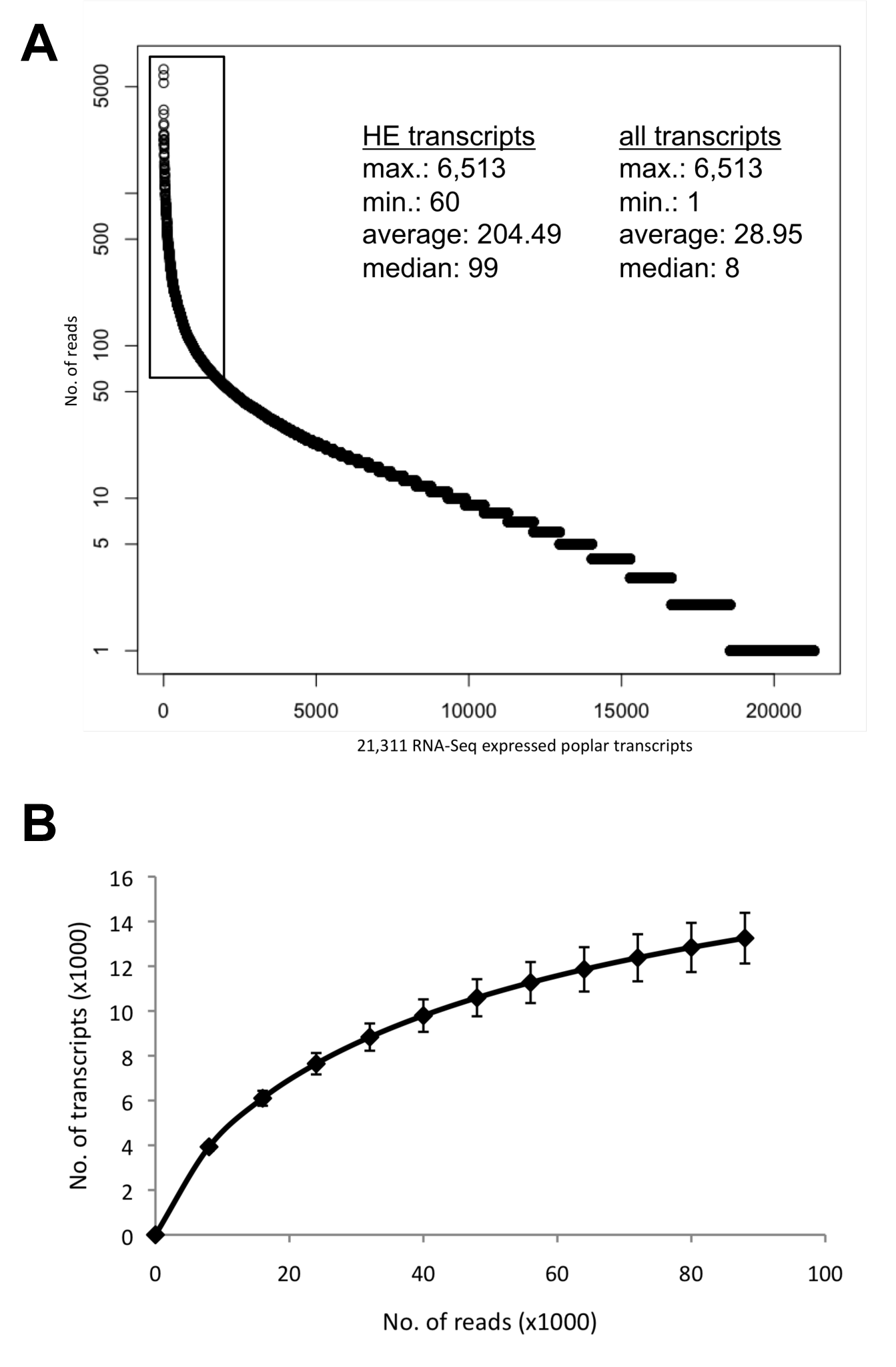

Supplement: Figure S4 — RNA-Seq coverage of the poplar transcriptome. (A) Distribution of RNA-Seq reads among the 21,311 expressed poplar transcripts. The black rectangle highlights the subset of 1,909 highly expressed (HE) transcripts considered for quantitative analyses. (B) Average saturation curve of the poplar transcriptome by RNA-Seq. (TIF) [file pone.0044408.s004.tif]

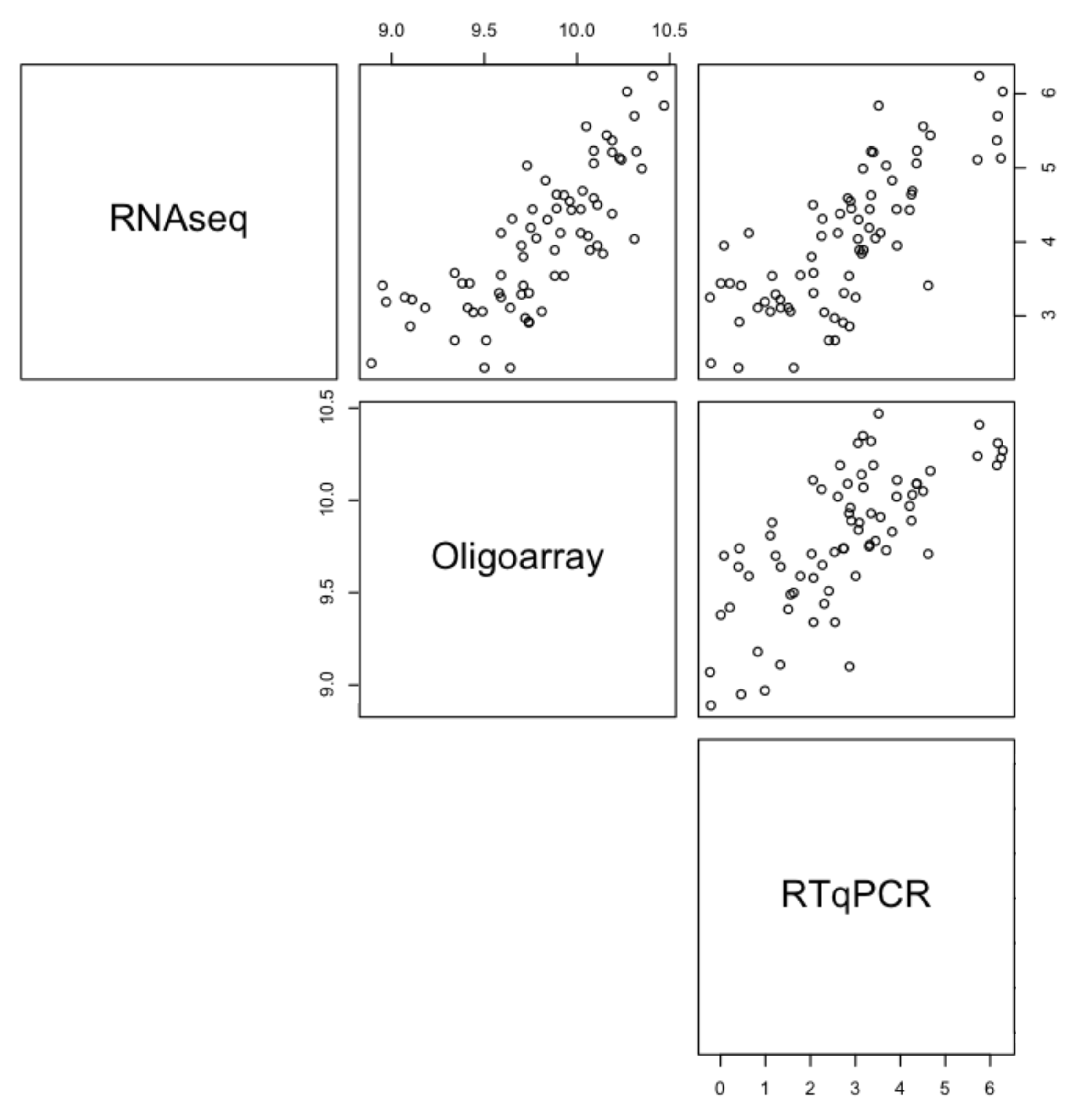

Supplement: Figure S5 — Correlation between RNA-Seq, oligoarrays and RT-qPCR normalized expression levels for 12 selected validated highly expressed (VHE) transcripts (listed in Table S2). (TIF) [file pone.0044408.s005.tif]

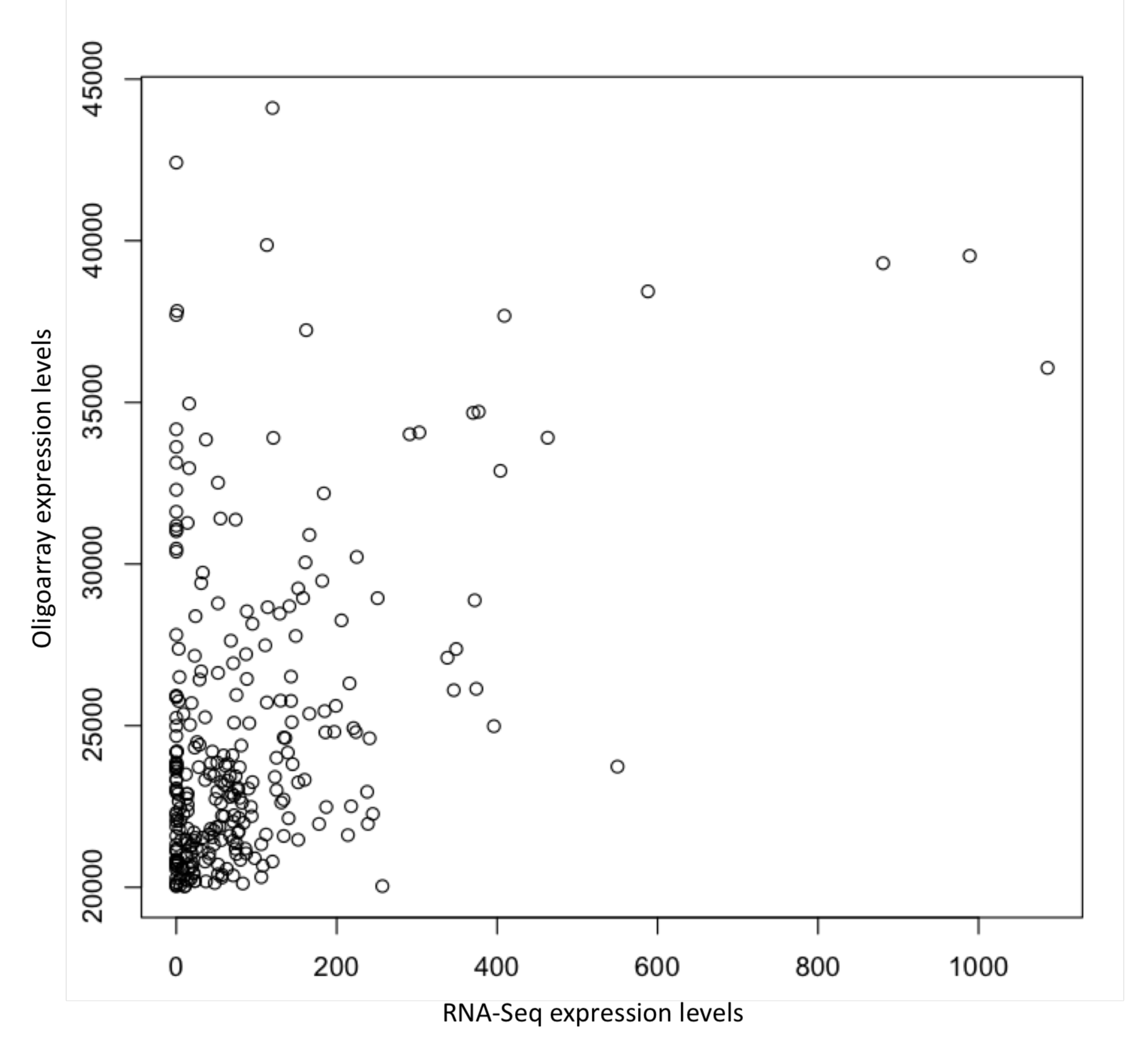

Supplement: Figure S6 — Dot-plot of the 285 most-expressed transcripts on oligoarrays (average expression values above 20,000) and corresponding RNA-Seq expression values. (TIF) [file pone.0044408.s006.tif]

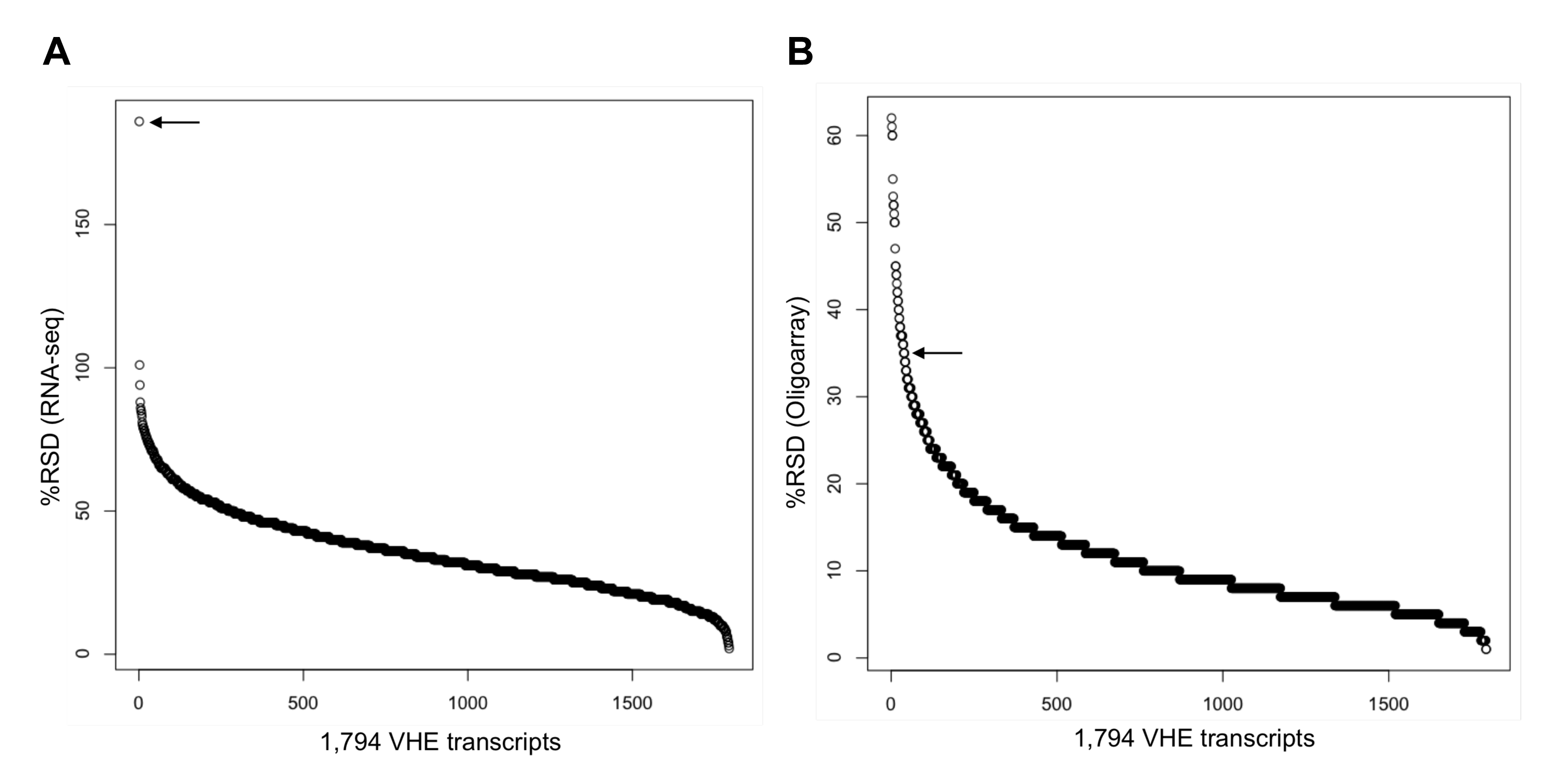

Supplement: Figure S7 — Percentage-relative standard deviation (%RSD) distributions. (A) %RSD for RNA-Seq expression levels of the 1,794 validated highly expressed (VHE) transcripts. (B) %RSD of oligoarray expression levels of the 1,794 VHE transcripts. Arrows in (A) and (B) indicate values of the fungal-induced sulfate transporter (PtSultr3;5, Poptr_0006s16150). (TIF) [file pone.0044408.s007.tif]

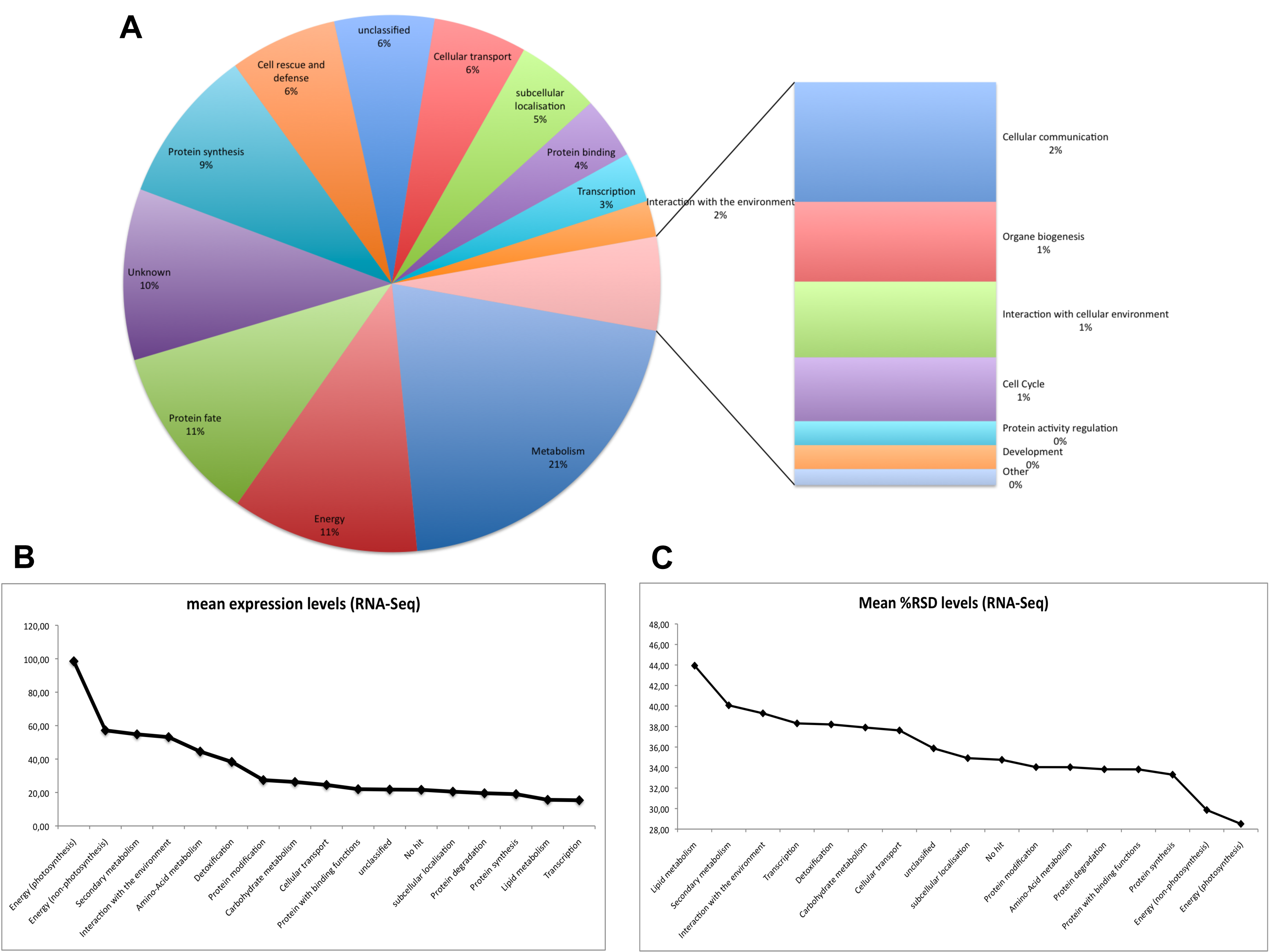

Supplement: Figure S8 — Functional Catalogue (FunCat) classification of the 1,794 validated highly expressed (VHE) transcripts. (A) Distribution of FunCat categories. Values represent percentage of the 1,794 VHE transcripts. (B) Average expression levels in FunCat categories. (C) Average percentage-relative standard deviation (%RSD) in FunCat categories. (TIF) [file pone.0044408.s008.tif]

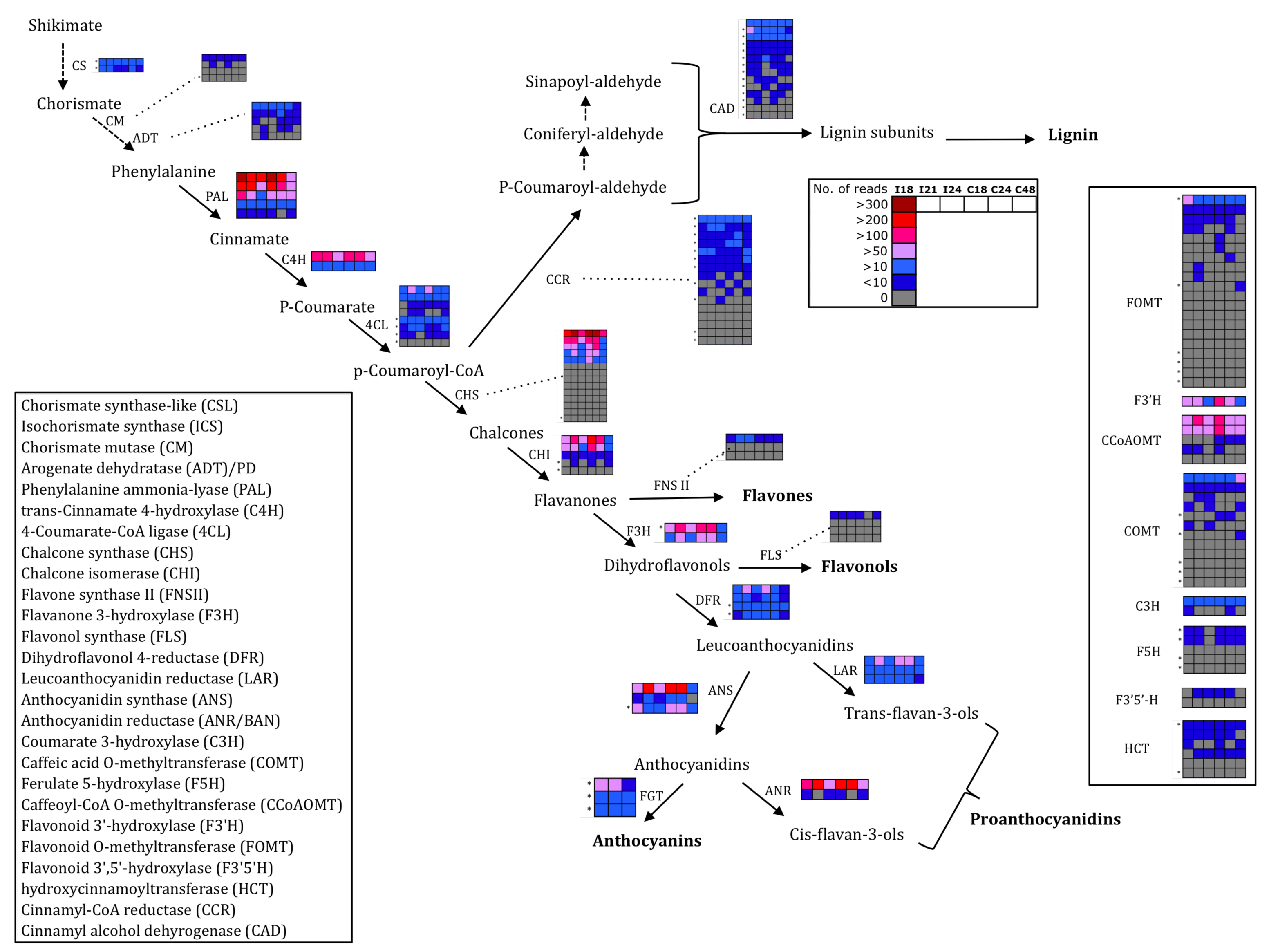

Supplement: Figure S9 — Transcriptional levels of the phenylpropanoid pathway transcripts in poplar leaves infected by M. larici-populina . (TIF) [file pone.0044408.s009.tif]

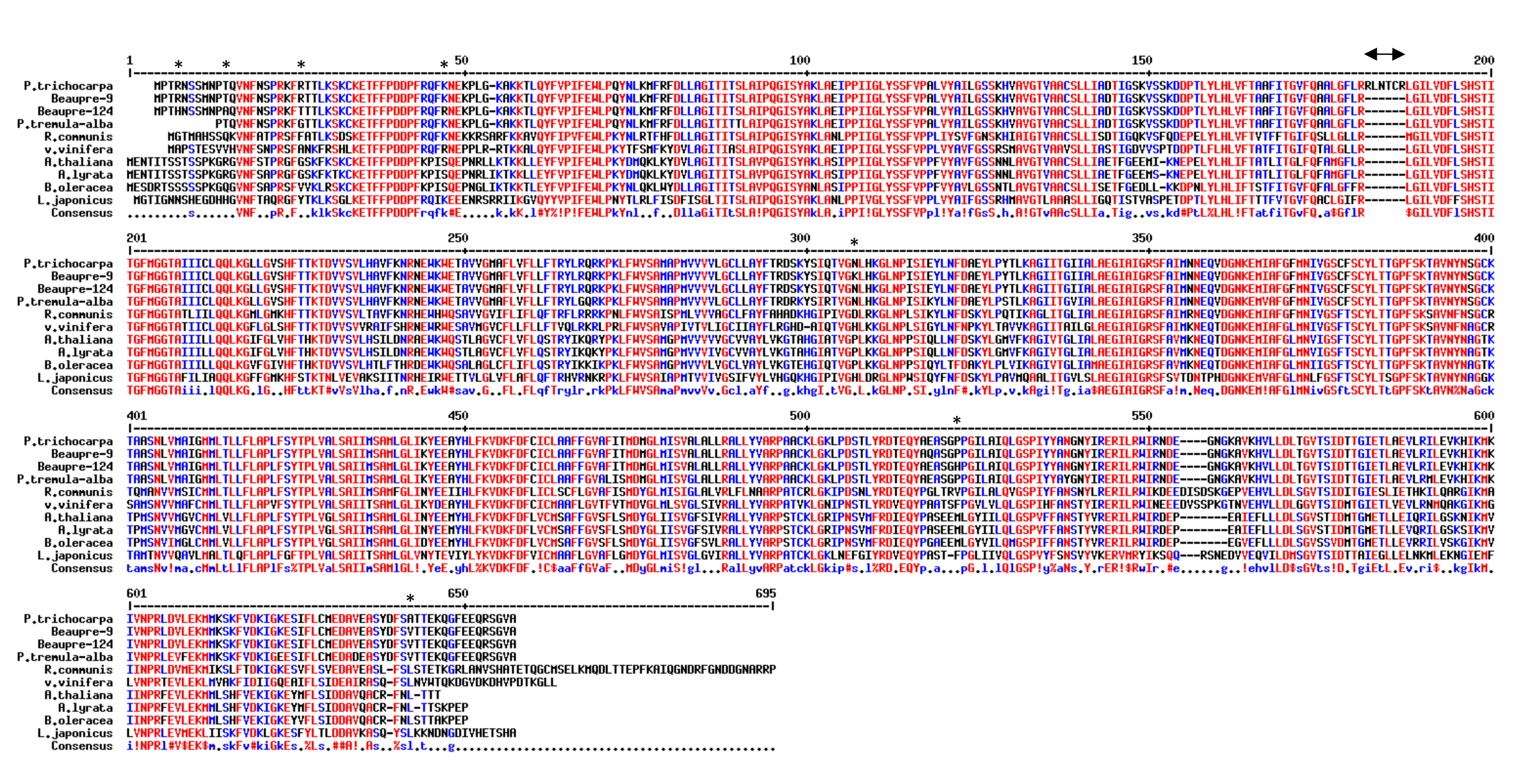

Supplement: Figure S10 — Alignment of selected plant sulfate transporters homologous to PtSultr3;5. Amino acid sequences of PtSultr3;5 (Populus trichocarpa, POPTR_0006s16150.1), Beaupré-9 (contig C48-lrc9, P. trichocarpa x Populus deltoides ‘Beaupré’), Beaupré-124 (contig C48-lrc-124, P. trichocarpa x P. deltoides ‘Beaupré’), AtSultr3;5 (Arabidopsis thaliana, At5g19600), NCBI ID 117557144 (PtaSultr3;5, Populus tremula x Populus alba), NCBI ID 255549068 (Ricinus communis), NCBI ID 225445290 (Vitis vinifera), NCBI ID 45720463 (Brassica oleracea), NCBI ID 297812143 (Arabidopsis lyrata) and SST1 (Lotus japonicus). Asterisks indicate polymorphic residues between ‘Beaupré’ alleles and the double-head arrow marks additional predicted amino acids in the P. trichocarpa ‘Nisqually-1’ genome discussed in Test S3. (TIF) [file pone.0044408.s010.tif]
