# Supplementary material for: RNA-Seq of Early-Infected Poplar Leaves by the Rust Pathogen Melampsora larici-populina Uncovers PtSultr3;5, a Fungal-Induced Host Sulfate Transporter
Source: PLoS One. 2012 Aug 30;7(8):e44408. doi: 10.1371/journal.pone.0044408 (PMC3431362; doi:10.1371/journal.pone.0044408)
Supplement: Text S1 — Uncorrelated oligoarray/RNA-Seq transcript expression. (DOC) [file pone.0044408.s013.doc]

**Text S1**. Uncorrelated oligoarray/RNA-Seq transcript expression.

Among the 285 most-expressed transcripts on oligoarray (mean expression level above 20,000), only 126 are validated highly expressed (VHE) transcripts and 54 were not supported at all by RNA-Seq (Fig. S6, Table S1). The lack of saturation for RNA-Seq could partially explain this observation, as more in-depth sequencing can provide a better correlation with oligoarray data **[40,68]**. However, a detailed analysis of the 54 transcripts not supported by RNA-Seq revealed that 35 of them correspond to genes with predicted alternative transcripts, of which at least one presents a strong expression by RNA-Seq (Table S1). For other transcripts without support in RNA-Seq data, most have a very close paralog exhibiting high expression level. Altogether, this supports the idea that probe-related aspecific hybridizations occur on oligoarrays for highly similar transcripts and account for the observed differences.
