# Supplementary material for: RNA-Seq of Early-Infected Poplar Leaves by the Rust Pathogen Melampsora larici-populina Uncovers PtSultr3;5, a Fungal-Induced Host Sulfate Transporter
Source: PLoS One. 2012 Aug 30;7(8):e44408. doi: 10.1371/journal.pone.0044408 (PMC3431362; doi:10.1371/journal.pone.0044408)
Supplement: Text S2 — Functional classification of the 1,794 poplar validated highly expressed (VHE) genes. (DOC) [file pone.0044408.s014.doc]

**Text S2**. Functional classification of the 1,794 poplar validated highly expressed (VHE) genes

A Functional Catalogue (FunCat) classification of the 1,794 poplar VHE genes has been performed and manually curated. The five most-important categories gathering 1,117 genes are metabolism, energy, protein fate, protein with unknown function, and protein synthesis (Fig. S8a). The energy category exhibited the highest levels of expression and the lowest %RSD, highlighting important and stable transcriptional activities related to energy biogenesis and conversion in poplar leaves (Fig. S8b and S8c). On the contrary, lipid and secondary metabolism categories presented the highest %RSD, likely mirroring more responsive and adaptive metabolism. The well characterized phenylpropanoid biosynthesis pathway in poplar **[69-70]** showed a highly transcriptionally active profile in infected leaves illustrating the benefit of using RNA-Seq data to identify active metabolic pathways (Fig. S9). Indeed, each step of this pathway from the amino acid phenylalanine to the final biogenesis of lignin, flavone, flavonol, anthocyanin and proanthocyanidin compounds is supported at least by one representative transcript, the most expressed being phenylalanine ammonia-lyases (PALs), chalcone synthases (CHSs) and anthocyanidin reductases (ANRs) (Fig. S9). However, contrasting with the transcriptional induction of this pathway in late compatible *Populus-Melampsora* interactions **[71-72]**, no significant up-regulation was observed here, highlighting once more the absence of a characterized host defense response at early stages of interaction.
