# Supplementary material for: RNA-Seq of Early-Infected Poplar Leaves by the Rust Pathogen Melampsora larici-populina Uncovers PtSultr3;5, a Fungal-Induced Host Sulfate Transporter
Source: PLoS One. 2012 Aug 30;7(8):e44408. doi: 10.1371/journal.pone.0044408 (PMC3431362; doi:10.1371/journal.pone.0044408)
Supplement: Text S3 — Interest and use of 454-sequences for complementary annotation of poplar and poplar rust genomes. (DOC) [file pone.0044408.s015.doc]

**Text S3**. Interest and use of 454-sequences for complementary annotation of poplar and poplar rust genomes

In addition to an accurate tool for measuring gene expression, RNA-Seq data also provide valuable sequence information for the exact determination of coding DNA sequences (CDS), the discovery of unpredicted genes, as well as the detection of alternative splicing variants. The contig-blast method primarily described often identified two contigs corresponding to a single poplar transcript (Table S1). Such twin contigs likely reflect divergent allelic versions in the *P. trichocarpa* x *P. deltoides* hybrid ‘Beaupré’ cultivar*.* Thus, for highly expressed genes, it was possible to detect allelic polymorphism in ‘Beaupré’. This is well exemplified with contigs C48-lrc9 (673 reads) and C48-lrc124 (234 reads) that both match to *PtSultr3;5* and present 7 non-synonymous polymorphic positions (Fig. S10). Moreover, alignment of both contigs with the predicted CDS in the *P. trichocarpa* ‘Nisqually-1’ genome revealed a mispredicted exon-intron structure resulting in 6 additional amino acids (Fig. S10). All these sequence informations (polymorphism and correction of exon-intron structures) were used for the design of highly specific primers for RT-qPCR with ‘Beaupré’ samples (Table S2). Otherwise, the read-mapping method using the PASA software identified 71 poplar model genes presenting alternative splicing events (data not shown). In addition, 3736 expressed loci were uncovered in the poplar genome sequence that might represent new formerly unpredicted genes (data not shown).

By the read-mapping method, PASA clustered 396 reads into 332 contigs assigned to 261 genes present in the actual *M. larici-populina* gene catalog. Interestingly, this method revealed the expression of 35 loci in the genome lacking gene annotation that might represent new coding sequences (data not shown). The detailed annotation of these loci is ongoing and will likely help in identifying putative new genes. Finally, the cDNA assembly annotation comparison with *M. larici-populina* genes and genome allowed 107 untranslated region additions, 15 genes extension and 22 internal gene structure rearrangements (data not shown)***.*** Since our data were obtained from two distinct strains (virulent 98AG31 vs. avirulent 93ID6), it was possible to notice polymorphic sites between allelic variants, including for potential candidate effectors (data not shown). Thus, such a transcript sequencing strategy from plant tissues infected by distinct strains could help identifying mutations that could relate to the pathogen virulence.
